# Costs of productivity loss up to two years after ischaemic stroke

**DOI:** 10.1007/s10198-025-01856-6

**Published:** 2025-10-21

**Authors:** Ilse Huijberts, Robert J. van Oostenbrugge, Wim H. van Zwam, Alida A. Postma, Florentina M.E. Pinckaers

**Affiliations:** 1 Department of Radiology and Nuclear Medicine, Maastricht University Medical Centre+, P. Debyelaan 25, Maastricht, 6229 HX The Netherlands; 2 Department of Neurology, Maastricht University Medical Centre+, Maastricht, The Netherlands; 3 Mental Health and Neuroscience Research Institute (MHENS), Maastricht University, Maastricht, The Netherlands; 4 Cardiovascular Diseases Research Institute (CARIM), Maastricht University, Maastricht, The Netherlands; 5 Care and Public Health Research Institute (CAPHRI), Maastricht University, Maastricht, The Netherlands

**Keywords:** Costs and cost analysis, Health economics, Productivity, Ischemic stroke, Modified rankin scale, I10, J24, I18

## Abstract

**Introduction:**

In the working population, diminished productivity increases the economic burden of ischaemic stroke. This study aimed to estimate costs of paid productivity losses per modified Rankin Scale (mRS) score at different time points up to two-years post-stroke.

**Patients and methods:**

Data from a multicentre cross-sectional study on post-stroke costs were utilised. Only patients with a pre-stroke professional activity were included in this study. Patients participated in a telephone mRS-interview and returned a questionnaire on paid productivity loss due to absenteeism and presenteeism in the preceding three months. Records were categorised into three-month, one-year, and two-year post-stroke time points based on the interview date. Costs of paid productivity loss were calculated using the human-capital approach (HCA) and friction cost method (FCM). Bootstrapping was combined with multiple imputation to derive mean costs per time point and mRS score (0, 1, 2, and 3–5).

**Results:**

Out of 1106 records, 280 respondents had a pre-stroke professional activity. Costs of paid productivity loss according to both the HCA and FCM ranged from €8980 (mRS 1) to €17,620 (mRS 3–5) in the first three months post-stroke. At one- and two years post-stroke, cost estimates according to the HCA were very similar to those at three months, while costs according to the FCM were significantly lower (0-775 euro at one year and 113–1884 euro at two years post-stroke).

**Discussion:**

Costs of productivity loss following ischaemic stroke in the working population are substantial and rise with higher mRS scores.

**Conclusion:**

These estimates may be used to inform health economic evaluations.

**Supplementary Information:**

The online version contains supplementary material available at 10.1007/s10198-025-01856-6.

## Introduction

Each year, over 7.6 million individuals experience an ischaemic stroke [1]. Over 58% of these cases manifest in individuals under the age of 70, and 11% are classified as young-strokes, affecting patients aged 15–49 [1, 2]. While the aging population in Europe results in an increase in the absolute number of ischaemic stroke, the most substantial rise in incidence is observed in young strokes. This is primarily attributable to risk factors such as obesity, diabetes and hypertension [3–6].

Ischaemic stroke in younger individuals results in a profound personal, societal and economic burden. Considering the economic burden, loss of productivity is increasingly recognised as a contributing factor. Productivity loss can be classified into paid work loss - which includes absenteeism (absence from work) and presenteeism (reduced productivity during work hours) - and unpaid work loss (e.g., voluntary work and household tasks) [7–9]. Various approaches exist for the evaluation of the costs of productivity loss, two prominent methods being the human-capital approach (HCA) and the friction-cost method (FCM) [9, 10]. The HCA adopts an employee-centric viewpoint, considering all unworked hours as losses [11]. In contrast, the FCM adopts a society-central viewpoint, measuring solely the hours lost within the friction period, which is the duration after which the absent employee is likely to have been replaced [9, 10].While the HCA tends to overestimate costs of productivity loss, the FCM potentially underestimates them as replacement employees trigger a chain of vacancies, each with their own friction period [11].

Though prognostic factors for successful return to work after stroke have been researched, the costs of productivity loss have received far less attention [12, 13]. Literature on this topic is scarce and is often dated or limited to retrospective, simulation- or registry-based studies [14, 15]. Previous studies also often omit presenteeism and/or unpaid work costs or restrict their analysis to patients who have resumed work [16–18]. The limited studies that do not have the aforementioned limitations either have a small patient sample size, a limited follow-up period, or have not analysed productivity losses per mRS-score, thereby complicating the integration of results into health economic evaluations [18, 19].

The aim of the current study is to assess the costs of paid and unpaid productivity losses per functional outcome on the mRS score, among ischaemic stroke patients with a pre-stroke professional activity, at different time points up to two years post-stroke, utilising both the HCA and FCM.

## Patients and methods

### Data source

Data were acquired from a cross-sectional study on health care and societal costs in ischaemic stroke patients [20]. Comprehensive methods have been described previously [20]. In brief, patients were retrieved from two sources: (1) a cross-sectional study at Maastricht University Medical Centre + involving stroke patients admitted between January 2019 and February 2022, and (2) the MR CLEAN-LATE trial, conducted from February 2018 and January 2022 across 18 stroke intervention centres in the Netherlands [21]. In the cross-sectional study, post-stroke cost estimates were collected at a single time point within two years post-stroke. The precise timing of this data collection (i.e. number of months post-stroke) varied between included patients. For the MR CLEAN-LATE trial, post-stroke cost assessment was added to the trial proceedings during the inclusion phase of the trial. The cost assessment could take place at three months, one year, and two years post-stroke. As a result, some patients were assessed at multiple time points. Only patients with a self-reported pre-stroke professional activity were included in the present study. Included patients participated in a telephone interview during which the mRS-score was assessed at a given timepoint up to two years post-stroke. Based on the interview date, patient records from both data sources were categorised into a time point: three months (3–6 months); one year (6–18 months); or two years (18–30 months) post-stroke. The telephone interview was combined with a written questionnaire on productivity losses. The data collection also included the assessment of patient characteristics such as education level, medical history and stroke characteristics.

### Patient outcomes

The mRS is a commonly used scale to evaluate functional outcome after ischaemic stroke, with values ranging from 0 (no symptoms) to 6 (death) [22]. A score of 1 indicates no significant disability, while 2 reflects limitations in daily activities such as the ability to work. Scores of 3 or higher indicate functional dependence. A trained researcher assessed the mRS during an interview with either the patient or their representative.

### Productivity assessment

A questionnaire that closely mirrored the structure and content of the iMTA Productivity Cost Questionnaire (iPCQ) was used to examine patient productivity losses in the preceding three months [8]. The questionnaires were sent by post or by mail to the patient or their representative. Absenteeism (hours of paid employment absence), presenteeism (hours of reduced productivity during work hours) and unpaid work losses (e.g., voluntary work and household tasks) were evaluated. Each questionnaire was validated and in case of missing or inconsistent data, the patient or representative was contacted by telephone. The questionnaire used is provided in Supplemental Appendix 1 (Online Supplementary Material).

### Missing data

Missing data occurred in baseline characteristics and unpaid work costs. The collective missingness rate for baseline patient characteristics was 0.5%. 18% of unpaid work cost estimates were missing as questions on unpaid work costs were not included in the first version of the cost questionnaire. There was no missing data in mRS scores and paid work costs.

### Productivity costing

The hourly cost of productivity loss was based on the Dutch Manual for Cost Analysis in Health Care Research [10]. The costs were indexed for the year 2022, resulting in a fixed hourly cost of €42,50 for paid work, and €17 for unpaid work.

### Statistical analysis and missing data

Crude data were used to describe baseline characteristics for all patients categorised by their post-stroke time point.

Mean costs of paid productivity loss were analysed using both the HCA and the FCM. Considering the FCM, a friction period of 90 days (three months) was used [10]. Multiple imputation was combined with bootstrapping to concurrently address missing data and non-normality. Multiple imputation was performed using Multiple Imputation by Chained Equations (MICE) [23]. The following variables were included in the imputation model: age; sex; baseline National Institutes of Health Stroke Scale (NIHSS) score; intravenous thrombolysis (IVT); endovascular treatment (EVT); discharge destination; residency at the time of the interview; interview mRS score and time point; anterior/posterior circulation stroke; education level; total healthcare costs and EQ-5D-5 L derived utilities. Logistic regression, ordered logit models and predictive mean matching were used as imputation methods. The number of iterations and imputations were set at 30 and 20, respectively.

A total of 1000 bootstrap samples were generated from the imputed dataset using non-parametric bootstrapping [24], after which mean and standard error estimates were derived for the costs of productivity loss at each time point and mRS category [24]. Patients with an mRS score of 3–5 were always combined into a single category in our analyses, as they constituted a relatively small part of the study population and their productivity loss was generally severe. Additional analyses for paid productivity loss were performed after correction for a 40-hour workweek.

Negative binomial regression analyses were performed to assess the association between costs of paid productivity loss and the mRS score at each time point. These analyses were deemed unfeasible for unpaid costs due to a paucity of data. Age, sex, education level and EVT were evaluated as predictors for return to work at all time points with the use of logistic regression (after correction for the mRS score).

Sensitivity analyses for paid productivity losses were conducted including solely patients who were still of working age according to the Dutch statutory retirement age (67 years) at their respective time point [25].

Statistical analyses were performed using R version 4.4.1 [26]. *Mice* version 3.16.0 was used for MICE [23]. *Boot* version 1.3.28 was used for bootstrapping.

## Results

### Patient characteristics

Out of a total of 1106 records, respondents of 280 records (25%) had paid work at the time of stroke onset and were included in this study. Of these records, 123 were derived from the MR CLEAN-LATE trial and 157 from the cross-sectional study. Thirty-one records (11%) were derived from patients who participated at multiple time points. Patient characteristics per time point are outlined in Table 1.

**Table 1:** Patient characteristics (*n* = 280)

	3 months (*n* = 65)	1 year (*n* = 102)	2 years (*n* = 113)
Demographic characteristics			
Age-median(IQR)	57(51–62)	60(54–63)	58(50–63)
Male sex-n(%)	39(60)	72(71)	78(69)
High education-n(%)	16(28)	35(35)	36(32)
Residency at interview-n(%)			
Home	56(86)	97(96)	105(93)
Nursing home	9(14)	4(4)	8(7)
mRS at interview-n(%)			
0	3(5)	9(9)	15(13)
1	5(8)	17(17)	20(18)
2	43(66)	64(63)	46(41)
3	5(8)	7(7)	17(15)
4	5(8)	5(5)	12(11)
5	4(6)	0(0)	3(3)
Medical history-n(%)			
Previous ischaemic stroke	6(9)	12(12)	9(8)
Pre-stroke mRS ≤ 2	65(100)	102(100)	113(100)
Stroke characteristics			
NIHSS-median(IQR)	6(3–12)	4(2–11)	4(2–9)
Anterior circulation-n(%)	60(92)	85(83)	95(85)
Endovascular treatment-n(%)	28(43)	35(34)	37(33)
Intravenous thrombolysis-n(%)	9(14)	35(34)	20(18)

^Abbreviations: *mRS*modified Rankin Scale, *NIHSS*National Institutes of Health Stroke Scale^

^Note: Based on crude data. If at least higher general secondary education was attended, education was classified as ’high’^

Before stroke, median weekly working hours were 36 (IQR 27–40) hours on 5 (IQR 4–5) working days. 160 patients (57%) worked at least 36 h per week. Pre-stroke working hours per mRS score and time point are presented in Supplemental Appendix 2 (Online Supplementary Material).

### Paid work costs

Mean costs according to the HCA and FCM at the three-month, one-year, and two-year post-stroke time points are presented in Tables 2, 3 and 4.

**Table 2:** Mean (SE) costs of productivity losses in the first 3 months post-stroke (*n* = 65)

	mRS 0 (*n* = 3)	mRS 1 (*n* = 5)	mRS 2 (*n* = 43)	mRS 3–5 (*n* = 14)
Paid work				
Presenteeism	0(0)	0(0)	72(37)	0(0)
Absenteeism	10,464(3851)	8980(3128)	13,812(1464)	17,620(1872)
Total costs	10,464(3851)	8980(3128)	13,884(1462)	17,620(1872)
Unpaid work				
Total costs	713(1438)	594(1046)	1635(817)	2081(1296)

^Abbreviations: *mRS*modified Rankin Scale^

^Note: For the first three months after a stroke, both the friction cost method and the human capital approach yield the same cost estimates (as the friction period was set at 90 days). Costs are in euros^

**Table 3:** Mean (SE) costs estimates of productivity losses in the previous three months at one year post-stroke (*n* = 102)

	mRS 0 (*n* = 9)	mRS 1 (*n* = 17)	mRS 2 (*n* = 64)	mRS 3–5 (*n* = 12)
Paid work-HCA				
Presenteeism	0(0)	31(24)	525(146)	0(0)
Absenteeism	604(310)	7215(2700)	10,014(1036)	13,608(2519)
Total costs	604(310)	7246(2697)	10,539(1017)	13,608(2519)
Paid work-FCM				
Presenteeism	0(0)	31(24)	525(146)	0(0)
Absenteeism	449(283)	131(100)	250(108)	0(0)
Total costs	449(283)	162(123)	775(208)	0(0)
Unpaid work				
Total costs	0(0)	108(83)	662(215)	1784(861)

^Abbreviations: *FCM*friction cost method, *HCA*human capital approach, *mRS*modified Rankin Scale^

^Note: Costs are in euros^

**Table 4:** Mean (SE) costs estimates of productivity losses in the previous three months at two years post-stroke (*n* = 113)

	mRS 0 (*n* = 15)	mRS 1 (*n* = 20)	mRS 2 (*n* = 46)	mRS 3–5 (*n* = 32)
Paid work-HCA				
Presenteeism	152(111)	581(373)	171(89)	113(86)
Absenteeism	2292(1317)	7317(2644)	12,330(1542)	21,640(1364)
Total costs	2443(1377)	7898(2603)	12,500(1522)	21,753(1347)
Paid work-FCM				
Presenteeism	152(111)	581(373)	171(89)	113(86)
Absenteeism	1732(1181)	0(0)	56(36)	0(0)
Total costs	1884(1194)	581(373)	227(101)	113(86)
Unpaid work				
Total costs	80(61)	0(0)	604(198)	1497(397)

^Abbreviations: *FCM*friction cost method, *HCA*human capital approach, *mRS*modified Rankin Scale^

^Note: Costs are in euros^

In the first three months post-stroke, total costs of paid productivity loss ranged from €8980 (mRS 1) to €17,620 (mRS 3–5). At one- and two years post-stroke, total costs of paid productivity loss in the previous three months according to the HCA ranged from €604 (mRS 0) to €13,608 (mRS 3–5) and from €2443 (mRS 0) to €21,753 (mRS 3–5), respectively. At the one-year time point, 0.0% (mRS 0), 0.4% (mRS 1), 5.0% (mRS 2), and 0.0% (mRS 3–5) of the costs were attributable to presenteeism. At the two-year time point, presenteeism contributed for 6.2% (mRS 0), 7.4% (mRS 1), 1.4% (mRS 2) and 0.5% (mRS 3–5) to the total costs. Costs according to the FCM in the preceding three months yielded lower estimates, ranging from €0 (mRS 3–5) to €775 (mRS 2) at one year and from €113 (mRS 3–5) to €1884 (mRS 0) at two years post-stroke.

Partly due to substantial differences in the number of hours worked per week between patients (Supplemental Appendix 2, Online Supplementary Material), the cost estimates in Tables 2, 3 and 4 varied across mRS categories and time points. After correction of paid productivity loss for a standard 40-hour workweek, the estimates demonstrate a more consistent increase across the mRS categories (Supplemental Appendix 3, Online Supplementary Material).

The mRS score was significantly associated with costs at each time point (Supplemental Appendix 4, Online Supplementary Material).

### Unpaid work costs

231 patients received the questionnaire on unpaid work issues. 68 of these patients (29%) reported having unpaid work issues in the preceding three months.

Costs resulting from unpaid productivity loss increased with each mRS score. Mean unpaid work cost estimates at the three-month, one-year, and two-year post-stroke time points are presented in Tables 2, 3 and 4. At three months post-stroke, costs of unpaid productivity loss ranged from €594 (mRS 1) to €2081 (mRS 3–5). At one- and two years post-stroke, unpaid work costs in the previous three months ranged from €0 (mRS 0) to €1784 (mRS 3–5), and from €0 (mRS 1) to €1497 (mRS 3–5), respectively.

### Predictors for return to work

At 3 months post-stroke, 21 patients (32%) had returned to work. At one- and two- years post stroke, 61 (60%) and 57 (50%) patients had returned to work, respectively. We found no association between age, female sex, high education or EVT and return to work (Supplemental Appendix 5, Online Supplementary Material).

### Sensitivity analyses

At stroke onset, 30 respondents of 280 records (11%) had already reached the statutory retirement age in the Netherlands. At the one- and two-year time points, an additional 8 and 9 respondents, respectively, reached this age. Paid cost estimates excluding these patients yielded similar results when compared to the main analyses (Supplemental Appendix 6, Online Supplementary Material).

## Discussion

In this study, we aimed to assess paid and unpaid productivity losses in ischaemic stroke patients at different time points up to two years post-stroke according to the HCA and FCM. Costs of both paid and unpaid productivity losses increase with higher mRS scores. The mRS was significantly associated with paid productivity losses at all time points.

Several studies have previously examined paid productivity loss in stroke patients. Overall, our findings on absenteeism and presenteeism are in line with Barral et al. [19], taking changes in hourly wages over the years and the difference in time window of the reported costs into account. An advantage of our study, however, is that compared to Barral et al., who described costs for mRS 0–2 vs. 3–5 [19], we were able to show separate estimates for each mRS category from 0 to 2 (e.g. one year; €604 [mRS 0], €7215 [mRS 1], €10014 [mRS 2]). As we found substantial differences between the cost estimates for mRS 0, 1 and 2, our results suggest that the usage of dichotomised mRS scores may lead to less accurate results when using these values in economic evaluations. Although we also grouped mRS scores of 3–5 due to the limited sample size in these categories, this may be less of an issue than grouping mRS 0–2, as substantial and similar productivity losses are expected across all categories in this group due to the severity of functional impairment. Barral et al. applied a friction period of 50 days for the FCM and considered 80% of productivity losses as actual costs. Using this approach, they estimated overall mean costs of productivity loss according to the FCM in the first year after stroke to be €3685 [19], which is comparable to our study. Other studies did not report productivity loss according to functional outcome or focused only on those patients who returned to work, making a direct comparison to our results unfeasible [16–18].

Considering unpaid work costs, previous studies primarily focused on indirect costs or unpaid work costs of caregivers of retired patients. In contrast, our study specifically addressed unpaid productivity loss in patients with a pre-stroke professional activity, providing a more comprehensive view of their productivity loss [18, 19]. Our estimates, along with the previous findings on unpaid and indirect costs in caregivers and retired patients, underscore that unpaid costs can be substantial and that their inclusion can have a profound effect on the results of cost effectiveness analyses [27].

Although we found no previous studies that investigated the association between EVT and return to work, a recent systematic review and meta-analysis including 39 studies identified male sex, white collar work, stroke severity and independence in activities of daily living as positive prognostic factors for return to work, and aphasia as a negative prognostic factor [12]. Our study did not identify significant predictors for return to work. However, these analyses were exploratory, and no definitive conclusions should be drawn from the results since the data were not powered for this purpose.

Economic evaluations are playing an increasingly important role in guiding the allocation of limited healthcare resources. Costs of both paid and unpaid productivity losses represent a substantial portion of the total societal costs of stroke, yet they are often underrepresented in economic evaluations. This omission risks underestimating the economic impact of interventions, especially with the rising incidence of stroke in working-age populations [3, 5, 6]. Previous studies have highlighted the economic burden of stroke, assessing healthcare costs and societal costs per mRS score [20, 28], but few have focused specifically on working-age stroke survivors. Including productivity losses in cost-effectiveness analyses provides a more comprehensive understanding of the economic value of treatments and can better inform healthcare resource allocation [27]. However, this is currently hindered by significant international differences in methods for incorporating, identifying, measuring, and valuing these costs [27]. Dutch guidelines recommend using the FCM due to its societal perspective [10], rather than the HCA, which tends to overestimate productivity loss by adopting an employee-centric viewpoint [19, 29]. Exploring the impact of both methods in research and using standardised questionnaires, such as the validated iPCQ (on which our questionnaire was based), could enhance the integration of productivity losses into economic evaluations across different countries.

### Strengths and limitations

An important strength of this study is that we were able to assess costs resulting from productivity loss by functional outcome, which makes the results easily applicable and useful for future research. Furthermore, the use of both the HCA and FCM methods makes the results more generalisable to the international differences of assessing productivity loss. We also used a questionnaire which was strongly based on the validated iPCQ to assess productivity loss. The generalisability of our results was increased by including patients across 18 different stroke centres in the Netherlands. Lastly, our study included patients at various time points up to two years post-stroke, which allows us to observe changes in productivity beyond the initial 90 days, when recovery may continue to influence patients’ ability to return to work or resume other productive activities.

Our study also has limitations. First, despite including 280 records (of 249 patients), the distribution across mRS groups was uneven, leading to small sample sizes in some categories. Mean working hours and cost estimates based on these small sample sizes may be less reliable and should be interpretated with caution. Furthermore, since 31/280 respondents participated at two different time points, this could reduce variability in the results. Also, the use of self-reported questionnaires may introduce recall bias. As we used both trial data and data from a cross-sectional study, our population may deviate somewhat from the general stroke population of patients under 65 years. However, the usage of trial data also enabled us to include patients across all interventional centres in the Netherlands, which enhances the generalisability of our findings. Furthermore, the MR CLEAN-LATE trial had broad and pragmatic inclusion criteria, limiting inclusion bias. The generalisability to non-trial populations was improved by incorporating cross-sectional data. As the data collection of both studies took place at the MUMC+, the used questionnaires and procedures were consistent between the two datasets. Considering the representativeness of the cohort, it is relevant to note that available patient characteristics presented in this study (regarding medical history and stroke characteristics) align with those of a previously reported young-stroke population in the Netherlands [30]. As our study was conducted solely in the Netherlands, the findings might be less generalisable to other countries. However, if the population and working conditions are considered comparable, our results could be applied to estimate productivity losses in different cost structures or currencies.

## Conclusion

Costs arising from both paid and unpaid productivity losses after ischaemic stroke are substantial, and the mRS is a major determinant of the amount of these costs. Our estimates on costs resulting from productivity loss in the working population may be used to inform economic evaluations using a societal cost perspective.

## Supplementary Information

Below is the link to the electronic supplementary material.

Supplementary Material 1 (PDF 237 KB)

## Data Availability

R-files and information on analytic methods can be shared on reasonable request. Underlying source data cannot be distributed publicly in accordance with the General Data Protection Regulation.

## References

[1] Feigin, V.L., et al.: World Stroke Organization (WSO): Global Stroke Fact Sheet 2022. Int J Stroke **17**, 18–29 (2022). 10.1177/17474930211065917 34986727 10.1177/17474930211065917

[2] GBD 2019 Stroke Collaborators: Global, regional, and National burden of stroke and its risk factors, 1990–2019: A systematic analysis for the global burden of disease study 2019. Lancet Neurol **20**, 795–820 (2021). 10.1016/S1474-4422(21)00252-0 34487721 10.1016/S1474-4422(21)00252-0 PMC8443449

[3] Bejot, Y., et al.: Epidemiology of stroke in Europe and trends for the 21st century. Presse Med **45**, e391–e398 (2016). 10.1016/j.lpm.2016.10.003 27816343 10.1016/j.lpm.2016.10.003

[4] Berkhemer, O.A., et al.: A randomized trial of intraarterial treatment for acute ischemic stroke. N Engl. J. Med. **372**, 11–20 (2015). 10.1056/NEJMoa1411587 25517348 10.1056/NEJMoa1411587

[5] Ekker, M.S., et al.: Stroke incidence in young adults according to age, subtype, sex, and time trends. Neurology **92**, e2444–e2454 (2019). 10.1212/WNL.0000000000007533 31019103 10.1212/WNL.0000000000007533

[6] Yahya, T., et al.: Stroke in young adults: Current trends, opportunities for prevention and pathways forward. American Journal of Preventive Cardiology **3**, 100085 (2020). 10.1016/j.ajpc.2020.100085 34327465 10.1016/j.ajpc.2020.100085 PMC8315351

[7] Posnett, J., Jan, S.: Indirect cost in economic evaluation: The opportunity cost of unpaid inputs. Health Econ. **5**, 13–23 (1996). 10.1002/(SICI)1099-1050(199601)5:1<13::AID-HEC182>3.0.CO;2-J 8653189 10.1002/(SICI)1099-1050(199601)5:1<13::AID-HEC182>3.0.CO;2-J

[8] Bouwmans, C., et al.: The iMTA productivity cost questionnaire: A standardized instrument for measuring and valuing Health-Related productivity losses. Value Health. **18**, 753–758 (2015). 10.1016/j.jval.2015.05.009 26409601 10.1016/j.jval.2015.05.009

[9] Koopmanschap, M.A., et al.: The friction cost method for measuring indirect costs of disease. J. Health Econ. **14**, 171–189 (1995). 10.1016/0167-6296(94)00044-5 10154656 10.1016/0167-6296(94)00044-5

[10] Hakkaart-van Roijen, L.H., et al.: Richtlijn voor het uitvoeren van economische evaluaties in de gezondheidszorg. Zorginstituut Nederland. (2015). https://www.zorginstituutnederland.nl/publicaties/publicatie/2024/01/16/richtlijn-voor-het-uitvoeren-van-economische-evaluaties-in-de-gezondheidszorg. Accessed 10 Mar 2024

[11] van den Hout, W.B.: The value of productivity: human-capital versus friction-cost method. Ann. Rheum. Dis. **69**, i89–91 (2010). 10.1136/ard.2009.117150 19995754 10.1136/ard.2009.117150

[12] Orange, C., et al.: Determinants of return to work after a stroke: A systematic review and Meta-analysis. Arch. Phys. Med. Rehabil. **105**, 359–368 (2024). 10.1016/j.apmr.2023.08.027 37797913 10.1016/j.apmr.2023.08.027

[13] van der Kemp, J., et al.: Return to Work after mild-to-moderate stroke: Work satisfaction and predictive factors. Neuropsychol. Rehabil. **29**, 638–653 (2019). 10.1080/09602011.2017.1313746 28441897 10.1080/09602011.2017.1313746

[14] Banefelt, J., et al.: Work productivity loss and indirect costs associated with new cardiovascular events in high-risk patients with hyperlipidemia: Estimates from population-based register data in Sweden. Eur. J. Health Econ. **17**, 1117–1124 (2016). 10.1007/s10198-015-0749-y 26607457 10.1007/s10198-015-0749-y PMC5080301

[15] Tan, E., et al.: The economic and health burden of stroke among younger adults in Australia from a societal perspective. BMC Public. Health **22**, 218 (2022). 10.1186/s12889-021-12400-5 35114974 10.1186/s12889-021-12400-5 PMC8811989

[16] Lekander, I., et al.: Relationship between functional disability and costs one and two years post stroke. PLoS One **12**, e0174861 (2017). 10.1371/journal.pone.0174861 28384164 10.1371/journal.pone.0174861 PMC5383241

[17] Fattore, G., et al.: The social and economic burden of stroke survivors in italy: A prospective, incidence-based, multi-centre cost of illness study. BMC Neurol **12**, 137 (2012). 10.1186/1471-2377-12-137 23150894 10.1186/1471-2377-12-137 PMC3536660

[18] Kotseva, K., et al.: Patient and caregiver productivity loss and indirect costs associated with cardiovascular events in Europe. European Journal of Preventive Cardiology **26**, 1150–1157 (2019). 10.1177/2047487319834770 30955367 10.1177/2047487319834770

[19] Barral, M., et al.: Patients’ productivity losses and informal care costs related to ischemic stroke: A French population-based study. Eur. J. Neurol. **28**, 548–557 (2021). 10.1111/ene.14585 33047452 10.1111/ene.14585

[20] Pinckaers, F.M.E., et al.: Cost and Utility Estimates per Modified Rankin Scale Score up to 2 Years Post Stroke: Data to Inform Economic Evaluations From a Societal Perspective. Value Health. **27**, 441–448 (2024). 10.1016/j.jval.2024.01.001 10.1016/j.jval.2024.01.001 38244981

[21] Olthuis, S.G.H., et al.: Endovascular treatment versus no endovascular treatment after 6–24 h in patients with ischaemic stroke and collateral flow on CT angiography (MR CLEAN-LATE) in the netherlands: A multicentre, open-label, blinded-endpoint, randomised, controlled, phase 3 trial. Lancet **401**(10385), 1371–1380 (2023). 10.1016/S0140-6736(23)00575-5 37003289 10.1016/S0140-6736(23)00575-5

[22] van Swieten, J.C., et al.: Interobserver agreement for the assessment of handicap in stroke patients. Stroke **19**, 604–607 (1988). 10.1161/01.STR.19.5.604 3363593 10.1161/01.str.19.5.604

[23] van Buuren, S., Groothuis-Oudshoorn, K.: Mice: Multivariate imputation by chained equations in R. Journal of Statistical Software **45**, 1–67 (2011). 10.18637/jss.v045.i03

[24] Marshall, A., et al.: Combining estimates of interest in prognostic modelling studies after multiple imputation: Current practice and guidelines. BMC Med. Res. Methodol **9**, 57 (2009). 10.1186/1471-2288-9-57 19638200 10.1186/1471-2288-9-57 PMC2727536

[25] Ministerie van Algemene Zaken. Wanneer gaat mijn AOW in? Rijksoverheid.nl: (2025). https://www.rijksoverheid.nl/onderwerpen/algemene-ouderdomswet-aow/vraag-en-antwoord/wanneer-gaat-mijn-aow-in

[26] R Core Team: R: A language and environment for statistical computing. R Foundation for Statistical Computing. https://www.R-project.org/. Accessed 10 Nov 2024

[27] Krol, M., Brouwer, W., Rutten, F.: Productivity costs in economic evaluations: past, present, future. Pharmacoeconomics **31**, 537–549 (2013). 10.1007/s40273-013-0056-3 23620213 10.1007/s40273-013-0056-3

[28] Dewilde, S., et al.: Modified Rankin scale as a determinant of direct medical costs after stroke. Int. J. Stroke **12**, 392–400 (2017). 10.1177/1747493017691984 28164742 10.1177/1747493017691984

[29] Jiang, S., et al.: Incorporating productivity loss in health economic evaluations: A review of guidelines and practices worldwide for research agenda in China. BMJ Glob Health **7**, e009777 (2022). 10.1136/bmjgh-2022-009777 35977755 10.1136/bmjgh-2022-009777 PMC9389102

[30] Brouwer, J., et al.: Endovascular thrombectomy in young patients with stroke: A MR CLEAN registry study. Stroke **53**, 34–42 (2022). 10.1161/STROKEAHA.120.034033 34872339 10.1161/STROKEAHA.120.034033

