# Supplementary material for: Costs of productivity loss up to two years after ischaemic stroke
Source: Eur J Health Econ. 2025 Oct 21;27(3):691–8. doi: 10.1007/s10198-025-01856-6 (PMC13190521; doi:10.1007/s10198-025-01856-6)
Supplement: Supplementary file 1 — Supplementary Material 1 (PDF 237 KB) [file 10198_2025_1856_MOESM1_ESM.pdf]

Online Supplementary Material

Costs of Productivity Loss up to Two Years after Ischaemic Stroke.

Ilse Huijberts, MD[1-4], Robert J. van Oostenbrugge, MD, PhD[2,4], Wim H. van Zwam, MD, PhD[1,4], Alida A. Postma, MD, PhD[1,3], Florentina M.E. Pinckaers, MD[1,4,5]

Author affiliations: [1]Department of Radiology and Nuclear Medicine, Maastricht University Medical Centre, Maastricht, The Netherlands; [2]Department of Neurology, Maastricht University Medical Centre, Maastricht, The Netherlands; [3]Mental Health and Neuroscience Research Institute (MHENS), Maastricht University, Maastricht, The Netherlands; [4]Cardiovascular Diseases Research Institute (CARIM), Maastricht University, Maastricht, The Netherlands; [5]Care and Public Health Research Institute (CAPHRI), Maastricht University, Maastricht, The Netherlands.

Corresponding author: Ilse Huijberts, Email: ilse.huijberts@mumc.nl, Adress: P. Debyelaan 25, 6229 HX Maastricht, The Netherlands. T: +31(0)43-387 2738

Journal name: The European Journal of Health Economics

Supplemental Appendix 1: Questionnaire on productivity loss

Disclaimer: the questionnaire below constitutes a translation of the Dutch questionnaire used in the data collection. The translation was done by the authors of this study for illustration purposes and is not the official English version of the iPCO (on which the used questionnaire was closely based).

Questions about work

Question 1 What were your daily activities before your stroke? Please mark what you did most of the time.

☐ I was in school, I was studying

☐ I worked as an employee

☐ I was an entrepreneur

☐ I was a housewife, househusband

☐ I was unemployed

☐ I was on disability leave for……….. %

☐ I was retired or on early retirement

☐ I did something else, namely, ………………………………………

……………………………………………………………………………

The following questions relate to your job. Specifically, work for which you are paid.

Question 2. Did you have a paid job before the stroke? If you were an entrepreneur before the stroke, you may also check 'yes'.

☐ No

☐ Yes

Did you check 'yes'? Then please proceed with the next question.
Otherwise, continue with question 13. *First, please read the explanation above question 13.*

Question 3. How many hours per week did you work before the stroke? Count only the hours for which you were paid.

……………….. hours

Question 4. On how many days per week did you work before the stroke?

……………..… days

Question 5. Did you (partially) return to work after the stroke?

☐ No

☐ Yes, and I have the same job as before the stroke

☐ Yes, and I have another job, namely

………………………………………………………………………………

Did you check 'yes'? Then please proceed with the next question.
Otherwise, continue with question 13. *First, please read the explanation above question 13.*

Question 6. When did you (partially) return to work after the stroke?

day	month	year
☐☐	☐☐	☐☐☐☐

Question 7. How many hours per week have you been working on average since then? Count only the hours for which you were paid.

……………….. hours

Question 8. On how many days per week have you been working on average since then?

…………....… days

Questions 9. Have you had to take sick leave in the past 3 months? For instance, due to having the flue. Or because you were unexpectedly hospitalized.

☐ No

☐ Yes, I have been absent for ……………………………… days

(*Please note: Only count the <u>work days</u>)*

Question 10. Were there days during which you experienced physical or psychological issues while working?

☐ No

☐ Yes

Did you check yes? Then please proceed with question 11 and 12.
Otherwise, continue with question 13. *First, please read the explanation above question 13.*

Question 11. On how many work days did you experience physical or psychological issues while working? *Only count the <u>work days</u>.*

……………… work days

Question 12. On the days you had these issues, you may not have been able to accomplish as much work as usual. On average, how much work could you do on these days? Please refer to the numbers below. A 10 means you could do just as much as usual on these days, while a 0 means that you could not do any work. Circle the appropriate number.

I could not do any work on these days	I could do approximately half of the work on these days	I could do just as much as usual on these days

0 1 2 3 4 5 6 7 8 9 10

> You may also experience physical or psychological issues when performing <u>unpaid work</u>. This may limit your ability to do unpaid work. For instance, you may not be able to take care of children or engage in volunteer work. Or you might be unable to go grocery shopping or work in the garden. The following questions are about these situations.

Question 13. Were there days when you could do less unpaid work due to physical or psychological issues? This question pertains to days within the past 3 months.

☐ No

☐ Yes

Did you check yes? Then please proceed with questions 14 and 15.

Otherwise, proceed to the end of the questionnaire.

Question 14. On how many days was this the case? Count the days within the past 3 months.

……..…. days

Question 15. Imagine that someone (for instance, your partner, family member or an acquaintance) had assisted you on these days and had done all the unpaid work you could not do. On average, how many hours per day do you think that person would have spent helping you on these days?

An average of …………… hours per day

Supplemental Appendix 2: Pre-stroke working hours

Table S1: Pre-stroke working hours categorised by mRS score and time point poststroke (n=280)

	**3 months (n=65)**	**1 year (n=102)**	**2 years (n=113)**
**Working hours per weekmedian(IQR)**	36 (28-40)	35 (24-40)	40 (32-40)
mRS 0	50 (41-50)	36 (32-38)	38 (34-40)
mRS 1	18 (15-32)	40 (32-55)	40 (24-40)
mRS 2	35 (28-40)	33 (22-40)	40 (30-40)
mRS 3-5	40 (36-40)	24 (14-36)	40 (35-40)

Abbreviations: mRS=modified Rankin Scale **Note**: Based on crude data.

Supplemental Appendix 3: Cost estimates corrected for a 40-hour work week

Table S2: Mean (SE) cost estimates of productivity losses in the first 3 months post-stroke (n=65)

	**mRS 0 (n=3)**	**mRS 1 (n=5)**	**mRS 2 (n=43)**	**mRS 3-5 (n=14)**
Presenteeism	0 (0)	0 (0)	117 (58)	0 (0)
Absenteeism	11087 (4759)	17618 (3227)	16275 (1338)	20504 (1218)
Total costs	11087 (4759)	17618 (3227)	16392 (1342)	20504 (1218)

Abbreviations: mRS=modified Rankin Scale. **Note**: Costs are corrected for a 40-hour work week. For the first three months after a stroke, both the friction cost method and the human capital approach yield the same cost estimates. Costs are in euros.

Table S3: Mean (SE) cost estimates of productivity losses in the previous three months at one year poststroke (n=102)

	**mRS 0 (n=9)**	**mRS 1 (n=17)**	**mRS 2 (n=64)**	**mRS 3-4 (n=12)**
**Paid work - HCA**				
Presenteeism	0 (0)	31 (24)	624 (164)	0 (0)
Absenteeism	1721 (1023)	6756 (2322)	13236 (1118)	19085 (1794)
Total costs	1721 (1023)	6787 (2319)	13861 (1074)	19085 (1794)
**Paid work - FCM**				
Presenteeism	0 (0)	31 (24)	624 (164)	0 (0)
Absenteeism	486 (310)	131 (100)	273 (113)	0 (0)
Total costs	486 (310)	162 (123)	897 (225)	0 (0)

Abbreviations: FCM=friction cost method, HCA=human capital approach, mRS=modified Rankin Scale. **Note**: Costs are corrected for a 40-hour work week. Costs are in euros.

Table S4: Mean (SE) cost estimates of productivity losses in the previous three months at two years post-stroke (n=113)

	**mRS 0 (n=15)**	**mRS 1 (n=20)**	**mRS 2 (n=46)**	**mRS 3-5 (n=32)**
**Paid work - HCA**				
Presenteeism	108 (76)	600 (375)	204 (99)	142 (108)
Absenteeism	3724 (1832)	8973 (2291)	13292 (1453)	21576 (333)
Total costs	3832 (1847)	9572 (2215)	13496 (1431)	21718 (277)
**Paid work - FCM**				
Presenteeism	108 (76)	600 (375)	204 (99)	142 (108)
Absenteeism	1846 (1216)	0 (0)	57 (37)	0 (0)
Total costs	1955 (1221)	600 (375)	261 (109)	142 (108)

Abbreviations: FCM=friction cost method, HCA=human capital approach, mRS=modified Rankin Scale. **Note**: Costs are corrected for a 40-hour work week. Costs are in euros.

Supplemental Appendix 4: Regression analysis paid productivity

Table S5: Effect estimates (95% CI) for the association of mRS with costs of paid productivity losses per time point

	**3 months (n=65)**	**1 year (n=102)**	**2 years (n=113)**
**mRS 1**	0.86 (0.84-0.87)	11.96 (11.64-12.30)	2.40 (2.38-2.42)
**mRS 2**	1.33 (1.31-1.34)	17.29 (16.83-17.76)	3.22 (3.19-3.25)
**mRS 3-5**	1.69 (1.67-1.71)	22.57 (21.96-23.19)	5.60 (5.55-5.65)

Abbreviations: mRS=modified Rankin Scale.

Note: The estimates are derived from negative binomial regression and can be interpreted on a multiplicative scale; for example, an estimate of 1.33 would indicate a 33% increase in costs. mRS 0 is the reference category. All estimates are statistically significant.

Supplemental Appendix 5: Predictors for return to work

Table S6: Predictors for return to work

	**3 months (n=65)**	**1 year (n=102)**	**2 years (n=113)**
**Returned to work – n(%)**	21 (32)	61 (60)	57 (50)
**Age, years**			
50-60	OR 0.90 (0.22 to 3.77)	OR 0.75 (0.17 to 3.24)	OR 1.21 (0.33 to 4.44)
>60	OR 0.62 (0.14 to 2.69)	OR 0.49 (0.11 to 2.07)	OR 0.70 (0.19 to 2.58)
**Female sex**	OR 0.41 (0.12 to 1.38)	OR 0.64 (0.24 to 1.74)	OR 0.61 (0.22 to 1.67)
**High education**	OR 0.77 (0.19 to 3.01)	OR 1.67 (0.65 to 4.30)	OR 2.25 (0.81 to 6.29)
**EVT**	OR 1.03 (0.34 to 3.16)	OR 0.42 (0.17 to 1.08)	OR 0.59 (0.21 to 1.67)

Abbreviation: Estimates are retrieved from logistic regression analyses and reported as odds ratios with 95% confidence intervals. EVT = endovascular treatment, OR = odds ratio. **Note**: regression analyses were adjusted for the mRS score. No estimates were statistically significant.

Supplemental Appendix 6: Paid cost estimates in patients of working age

Table S7: Mean (SE) cost estimates of productivity losses during the first 3 months poststroke in patients of working age (n=61)

	mRS 0 (n=3)	mRS 1 (n=3)	mRS 2 (n=41)	mRS 3-5 (n=14)
Presenteeism	0 (0)	0 (0)	76 (39)	0 (0)
Absenteeism	10464 (3851)	8834 (4826)	14376 (1468)	17620 (1872)
Total costs	10464 (3851)	8834 (4826)	14452 (1465)	17620 (1872)

Abbreviations: mRS=modified Rankin Scale. **Note**: For the first three months after a stroke, both the friction cost method and the human capital approach yield the same cost estimates (as the friction period was set at 90 days). Costs are in euros. Working age was determined based on the statutory retirement age in the Netherlands for each individual patient (67 years).

Table S8: Mean (SE) cost estimates of productivity losses in the previous three months at one year post-stroke in patients of working age (n=82)

	mRS 0 (n=6)	mRS 1 (n=10)	mRS 2 (n=56)	mRS 3-5 (n=10)
**Paid work - HCA**				
Presenteeism	0 (0)	53 (41)	589 (162)	0 (0)
Absenteeism	682 (414)	4341 (2802)	9714 (1123)	15930 (2433)
Total costs	682 (414)	4395 (2807)	10304 (1102)	15930 (2433)
**Paid work - FCM**				
Presenteeism	0 (0)	53 (41)	589 (162)	0 (0)
Absenteeism	682 (414)	226 (171)	277 (121)	0 (0)
Total costs	682 (414)	280 (212)	866 (230)	0 (0)

Abbreviations: FCM=friction cost method, HCA=human capital approach, mRS=modified Rankin Scale. **Note**: Costs are in euros. Working age was determined based on the statutory retirement age in the Netherlands for each individual patient (67 years).

Table S9: Mean (SE) cost estimates of productivity losses in the previous three months at two years post-stroke in patients of working age (n=90)

	mRS 0 (n=14)	mRS 1 (n=12)	mRS 2 (n=39)	mRS 3-5 (n=25)
**Paid work - HCA**				
Presenteeism	162 (119)	1044 (627)	171 (98)	149 (111)
Absenteeism	2452 (1421)	7382 (3332)	12508 (1738)	21495 (1546)
Total costs	2614 (1486)	8426 (3236)	12679 (1714)	21644 (1520)
**Paid work - FCM**				
Presenteeism	162 (119)	1044 (627)	171 (98)	149 (111)
Absenteeism	1853 (1272)	0 (0)	66 (43)	0 (0)
Total costs	2015 (1288)	1044 (627)	238 (114)	149 (111)

Abbreviations: FCM=friction cost method, HCA=human capital approach, mRS=modified Rankin Scale. **Note**: Costs are in euros. Working age was determined based on the statutory retirement age in the Netherlands for each individual patient (67 years).
